# Supplementary material for: Human MIKO-1, a Hybrid Protein That Regulates Macrophage Function, Suppresses Lung Fibrosis in a Mouse Model of Bleomycin-Induced Interstitial Lung Disease
Source: Int J Mol Sci. 2022 Aug 26;23(17):9669. doi: 10.3390/ijms23179669 (PMC9456133; doi:10.3390/ijms23179669)
Supplement: Supplementary file 1 [file ijms-23-09669-s001.zip › Figure S1.pdf]

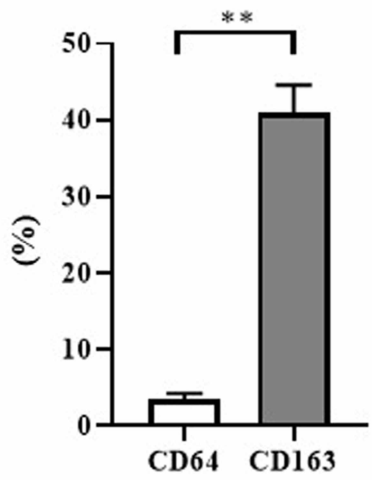

Figure S1. Cell surface antigens of thioglycolate-induced murine peritoneal macrophages. Expression of CD64 and CD163. Data are shown as mean ± SD (N = 6). ** $p < 0.01$, significant difference between the linked groups.
