# Supplementary material for: Human MIKO-1, a Hybrid Protein That Regulates Macrophage Function, Suppresses Lung Fibrosis in a Mouse Model of Bleomycin-Induced Interstitial Lung Disease
Source: Int J Mol Sci. 2022 Aug 26;23(17):9669. doi: 10.3390/ijms23179669 (PMC9456133; doi:10.3390/ijms23179669)
Supplement: Supplementary file 1 [file ijms-23-09669-s001.zip › Tables S1 and S2.pdf]

Table S1. Primers used in the qPCR analyses in vitro.

Gene	Forward (5' to 3')	Reverse (5' to 3')
CD64	CTTCTCCTTCTATGTGGGCAGT	GCTACCTCGCACCAGTATGAT
CD163	TCACTCCTGGGCTGCACGTAAAC	GATGTTATTTGCCATACAGGAGAATTG
IL-12	AAGATGAAGGAGACAGAG	CATTGGACTTCGGTAGAT
IL-10	GTGGAGCAGGTGAAGAGTGA	TTCATGGCCTTGTAGACACCT
GAPDH	ACAATGAATACGGCTACAG	GGTCCAGGGTTTCTTACT

Table S2. Primers used in the qPCR analyses in vivo.

Gene	Forward (5' to 3')	Reverse (5' to 3')
TNF-α	ACCTTGTTGCCTCCTCTT	GTTCAGTGATGTAGCGACAG
IL-6	AAATGAGAAAAGAGTTGTG	TTTGTATCTCTGGAAGTTT
IL-1β	GATACCACTCCCAACAGA	GCCATTGCACAACTCTTT
F4/80	TGTCTGAAGATTCTCAAAACATGGA	TGGAGCTTCATAGTTGTAAGGCA
MMP-9	CGATTCCAAACCTTCAAA	GCAAGTCTTCAGAGTAGT
TIMP-1	AAGATGACTAAGATGCTAA	GATGAGAAACTCTTCACT
GAPDH	ACAATGAATACGGCTACAG	GGTCCAGGGTTTCTTACT
